# Fragile X mental retardation protein expression in Alzheimer’s disease

**DOI:** 10.3389/fgene.2014.00360

**Published:** 2014-10-21

**Authors:** Abigail J. Renoux, Nicholas M. Carducci, Arya A. Ahmady, Peter K. Todd

**Affiliations:** ^1^Department of Molecular and Integrative Physiology, University of Michigan, Ann Arbor, MI, USA; ^2^Department of Neurology, University of Michigan, Ann Arbor, MI, USA

**Keywords:** fragile X, FMRP, Alzheimer’s disease, APP, mouse models

## Abstract

The *FMR1* protein product, FMRP, is an mRNA binding protein associated with translational inhibition of target transcripts. One FMRP target is the amyloid precursor protein (APP) mRNA, and APP levels are elevated in *Fmr1* KO mice. Given that elevated APP protein expression can elicit Alzheimer’s disease (AD) in patients and model systems, we evaluated whether FMRP expression might be altered in Alzheimer’s autopsy brain samples and mouse models compared to controls. In a double transgenic mouse model of AD (APP/PS1), we found no difference in FMRP expression in aged AD model mice compared to littermate controls. FMRP expression was also similar in AD and control patient frontal cortex and cerebellum samples. Fragile X-associated tremor/ataxia syndrome (FXTAS) is an age-related neurodegenerative disorder caused by expanded CGG repeats in the 5′ untranslated region of the *FMR1* gene. Patients experience cognitive impairment and dementia in addition to motor symptoms. In parallel studies, we measured FMRP expression in cortex and cerebellum from three FXTAS patients and found reduced expression compared to both controls and Alzheimer’s patient brains, consistent with animal models. We also find increased APP levels in cerebellar, but not cortical, samples of FXTAS patients compared to controls. Taken together, these data suggest that a decrease in FMRP expression is unlikely to be a primary contributor to Alzheimer’s disease pathogenesis.

## INTRODUCTION

Fragile X-associated disorders result from intergenerational instability in a CGG microsatellite repeat expansion located in the 5′ untranslated region (UTR) of the fragile X mental retardation (*FMR1*) gene. In the general population, the mean repeat length is 30 CGG repeats with a range from 4 to 55 (Strom et al., 2007). When this repeat expands beyond this normal range, it can cause symptoms associated with fragile X spectrum disorders (Renoux and Todd, 2012; Nelson et al., 2013). Greater than 200 CGG repeats elicits transcriptional silencing of the *FMR1* locus, with absent or markedly reduced FMR1 mRNA and FMR protein (FMRP) production (Bagni and Oostra, 2013). These large expansions result clinically in fragile X syndrome (FXS), which is the most common monogenic cause of autism and intellectual disability (Rogers et al., 2001; Hernandez et al., 2009).

In contrast, repeat expansions in the “premutation” range between 55 and 200 CGG repeats cause a distinct set of human disorders, including fragile X-associated tremor/ataxia syndrome (FXTAS) and fragile X-associated Premature Ovarian Insufficiency (FXPOI; Berry-Kravis et al., 2007; Hagerman, 2013; Nelson et al., 2013). FXTAS is an age-related neurodegenerative disorder characterized by gait difficulties, action tremor, and variably present Parkinsonism, dysautonomia, and dementia (Berry-Kravis et al., 2007). Unlike the scenario in FXS, premutation sized CGG repeats elicit enhanced *FMR1* transcription through alterations in the local chromatin structure (Tassone et al., 2000a, 2007; Todd et al., 2010; Hagerman and Hagerman, 2013). However, this increase in FMR1 mRNA is paradoxically associated with a reduction in total and activity-dependent FMRP expression (Tassone et al., 2000b; Kenneson et al., 2001; Entezam et al., 2007; Qin et al., 2011; Iliff et al., 2013; Ludwig et al., 2014; Pretto et al., 2014). This decrease in FMRP likely derives from an alteration in FMR1 mRNA translational efficiency, where the repeat forms a hairpin secondary structure that impairs ribosomal scanning (Feng et al., 1995; Kenneson et al., 2001; Primerano et al., 2002; Chen et al., 2003; Ludwig et al., 2011). Research into the mechanisms of neurodegeneration in FXTAS has largely focused on how the FMR1 mRNA might elicit a gain of function toxicity, either through sequestration of RNA binding proteins or through triggered aberrant translation through the repeat of aggregate prone proteins that underlie the intranuclear inclusions observed in patients (Jin et al., 2003, 2007; Hagerman and Hagerman, 2004, 2013; Iwahashi et al., 2006; Sofola et al., 2007; Sellier et al., 2010, 2013; Todd and Paulson, 2010; Renoux and Todd, 2012; Todd et al., 2013). However, more recent work has begun to explore whether a reduction in FMRP expression might contribute to aspects of disease pathogenesis in these individuals (Iliff et al., 2013; Ludwig et al., 2014; Pretto et al., 2014; Renoux et al., 2014; von Leden et al., 2014).

The absence of FMRP causes the cognitive impairment seen in FXS and may contribute to some of the symptoms observed in FXTAS. A significant body of work has explored the normal functions of FMRP (O’Donnell and Warren, 2002; Santoro et al., 2012; Wang et al., 2012). FMRP is an RNA binding protein found associated with poly-ribosome complexes in soma and synapses (Tamanini et al., 1996; Willemsen et al., 1996; Feng et al., 1997; Zalfa et al., 2003; Darnell et al., 2005, 2011; Ascano et al., 2012; Chen et al., 2014). It is normally phosphorylated, and upon metabotropic glutamate receptor (mGluR) activation is dephosphorylated to allow its associated transcripts to be translated (Ceman et al., 2003; Bear et al., 2004; Weiler et al., 2004; Muddashetty et al., 2007; Narayanan et al., 2008; Nalavadi et al., 2012). This allows FMRP to participate in temporal and spatial control of activity-dependent translation. In an effort to understand how reduced levels of FMRP may alter synaptic function, many groups have identified possible FMRP target transcripts. One transcript associated with FMRP is the amyloid precursor protein (APP) mRNA (Westmark and Malter, 2007; Lee et al., 2010). High-throughput sequencing of RNAs isolated by cross-linking immunoprecipitation (HITS-CLIP and PAR-CLIP) analysis on FMRP-associated transcripts identified APP mRNA (Darnell et al., 2011; Ascano et al., 2012). This interaction appears to play a role in regulating APP translation, as APP synthesis in response to mGluR activation is increased in control mice (Westmark and Malter, 2007). Moreover, a mouse model of FXS which lacks FMRP (*Fmr1* KO) exhibit higher basal levels of APP, and of the pathogenic product of APP cleavage, β-amyloid (Aβ), and FXS patients show abnormal Aβ levels in plasma and brain tissues (Westmark and Malter, 2007; Westmark et al., 2011). Overexpression of APP in *Fmr1* KO mice increases mortality and seizure susceptibility (Westmark et al., 2008). Conversely, genetic reduction of APP in the *Fmr1* KO mouse improved seizure activity, anxiety-associated behavior, spine morphology, and altered mGluR-dependent long-term depression (LTD), indicating a role for Aβ expression levels in FXS pathology (Westmark et al., 2011).

As there is evidence indicating FMRP participates in regulating APP production and that FMRP and APP interact genetically, we sought to explore the possibility that reduced FMRP levels may contribute to increased APP and Aβ levels in AD mouse models and spontaneous cases of AD. This is especially relevant given evidence for decreased FMRP expression with age in mouse models (Singh et al., 2007; Iliff et al., 2013; Gaur and Prasad, 2014; Ludwig et al., 2014). Impaired FMRP expression in older individuals could lead to increased basal APP translation, increasing the amyloidogenic burden and thus serving as a contributor to AD pathogenesis. We sought to test this hypothesis by measuring cortical FMRP levels by western blot and immunohistochemistry in a double transgenic AD mouse model (APP/PS1; Haass et al., 1995; Prihar et al., 1999). We also measured FMRP immunoreactivity in human cortex and cerebellum from control and confirmed AD samples. Concurrently, we included samples from three FXTAS patients who exhibited reduced FMRP levels. We found similar FMRP expression in AD model mice and AD human samples. We further examined APP expression in FXTAS patient samples, and found a selective increase in cerebellar lysates, but not in frontal cortex or in CGG KI model mice. Taken together, our data suggest that impaired FMRP expression is unlikely to contribute significantly to end-stage Alzheimer’s disease (AD) pathogenesis.

## MATERIALS AND METHODS

### MICE

Animal use followed NIH guidelines and was in compliance with the University of Michigan Committee on Use and Care of Animals. *Fmr1* KO (Bakker et al., 1994) and CGG KI (Entezam et al., 2007) mice were genotyped as described previously (Iliff et al., 2013; Renoux et al., 2014). APP/PS1 mouse (Haass et al., 1995; Prihar et al., 1999) genotypes were confirmed with western analysis for the human APP transgene (clone 6E10 1:2000; Millipore).

### PATIENT DONOR SAMPLES

All human tissues were obtained and distributed under oversight by appropriate institution specific review boards. Frontal cortex and cerebellar tissue from 10 control and 10 clinically probable AD patients were obtained from the University of Michigan Alzheimer’s Disease Brain Bank. All AD cases were confirmed at autopsy. Brain tissues from two previously described FXTAS patients (Louis et al., 2006; Todd et al., 2013) and an additional clinically definite FXTAS patient were used as controls for reduced FMRP expression. CGG repeat size was determined in FXTAS patients by DNA isolation followed by PCR using C and F primers. See Table 1 for post-mortem interval (PMI), age, and sex of each individual.

**Table 1:** **Patient donor information.**

Diagnosis	Sex	Age	PMI (hours)	FMR1 repeats
Control	F	82	21	n.d.
Control	M	39	22	n.d.
Control	M	47	23	n.d.
Control	M	69	24	n.d.
Control	M	72	23	n.d.
Control	F	86	18	n.d.
Control	F	87	9	n.d.
Control	F	74	6	n.d.
Control	M	59	12	n.d.
Control	M	81	6	n.d.
AD	F	78	2	n.d.
AD	M	69	12	n.d.
AD	F	75	24	n.d.
AD	F	66	9	n.d.
AD	M	82	9	n.d.
AD	M	73	3	n.d.
AD	F	86	15	n.d.
AD	M	80	21	n.d.
AD	F	75	24	n.d.
AD	M	69	13	n.d.
FXTAS	M	78	3.5	90 CGGs
FXTAS	M	74	3	102 CGGs
FXTAS	M	80	6.5	116 blood/180 cheek

Diagnosis, age at death, post-mortem interval (PMI) in hours for all patients, and FMR1 5′ UTR CGG length for FXTAS patients included above. n.d. indicates not determined.

### WESTERN BLOT ANALYSIS

Western blotting was performed as described previously (Iliff et al., 2013). Briefly, brain tissue samples (cerebral cortex and subcortical regions from mice, or frontal cortex from human, and cerebellum) were homogenized in RIPA buffer containing Complete Mini protease inhibitor cocktail (Roche). Samples were sonicated and centrifuged, and total protein content of the supernatant measured using a DC Protein assay (Bio-Rad). Equal amounts of protein were mixed with 6 × Laemmli buffer and boiled for 5 min before separation on 8% polyacrylamide gels. Gels were transferred and blocked with Tris-buffered saline containing 0.1% Triton-X (TBST) and 5% non-fat milk for 60 min at room temperature (RT), and incubated with an antibody against FMRP (Millipore mouse monoclonal 1C3 1:1000 or Abcam rabbit polyclonal 17722, 1:1000), or against the C-terminus of APP (Invitrogen 51-2700, 1:500) and β-tubulin (University of Iowa’s Developmental Studies Hybridoma Bank E7, 1:5000) overnight at 4°C. Blots were incubated with corresponding fluorescent secondary antibody (1:15000; IRDye^®^ 680RD or 800CW, LI-COR) for 60 min at RT, and imaged with the Odyssey^®^ Imaging System (LI-COR).

Band intensity was quantified in the linear range with densitometry using LI-COR Image Studio^TM^ Software. Experiments were performed in technical triplicate, and FMRP/tubulin or APP/tubulin ratios to two control samples included in every experiment were combined. These ratios were averaged, normalized to control values for each experiment, and expressed as %control in experiments.

### IMMUNOHISTOCHEMISTRY

Antibody control experiments were performed on mice aged 50–75 weeks (*n* = 6 WT, *n* = 5 CGG KI, *n* = 1 *Fmr1* KO), and experimental analysis on 80- to 90-week-old mice (*n* = 2 WT, *n* = 3 APP/PS1) which were anesthetized with 0.2 mg ketamine/20 µg xylazine per kilogram prior to transcardial perfusion with 15–20 mL phosphate buffered saline (PBS) and 15–20 mL 4% paraformaldehyde (PFA) in PBS. Brains were dissected, fixed overnight in 4% PFA, and then sunk in 30% sucrose in PBS at 4°C. Brains were sectioned at 30 µm and placed in a cryostorage solution of 30% sucrose/33.33 % ethylene glycol/0.05 M PBS until needed.

Prior to staining, slices were rotated in PBS overnight at 4°C to remove cryostorage solution, then basic antigen retrieval was performed by placing the slices in 0.01 M sodium citrate (pH 8.5) at 80°C for 10 min followed by three 5-min washes in PBS. The slices were then placed in 1% H_2_O_2_ in Tris to block endogenous peroxidases. Slices were permeabilized in 0.1% Triton-X/.05% bovine serum albumin (BSA) in Tris for 30 min at RT and blocked in 5% normal goat serum (NGS) for 1 h at RT. Slices were then incubated overnight in anti-FMRP antibody (1:3500). Following two washes, slices were incubated in horseradish peroxidase-conjugated secondary antibody (1:1000) in 5% NGS in Tris. Prior to peroxidase development, slices were treated with the Vectastain ABC kit (Vector) to increase diaminobenzidine (DAB) visibility. Following washes, slices from all genotypes were placed simultaneously in ImmPACT DAB solution (Vector) for 10–15 s until they just started to turn brown. Following washes, slices were mounted, allowed to dry overnight then either counterstained in Gill’s 1:2 hematoxylin for 45–60 s, or immediately dehydrated in an alcohol gradient and mounted.

### MICROSCOPY

A slide scanning microscope (Zeiss) was used to image all DAB stained tissue. Fields of view were selected in the hippocampus and cortex to be easily reproducible across multiple sections. Images were taken using the same exposure settings for all genotypes.

### STATISTICAL ANALYSIS

All values are expressed as the mean ± standard error of the mean. Western blot immunoreactivity for each sample was measured in technical triplicate, and combined as a ratio of %control for each blot. These combined values were averaged, and compared using a Student’s *t*-test, with significance indicated by a *P*-value < 0.05.

FMRP expression compared to age, PMI, and pH were evaluated using a Pearson’s correlation coefficient on combined cortical and cerebellar FMRP values from either control and AD samples, or combined control and FXTAS cortical and cerebellar values, with significance indicated by *P* < 0.05. Comparison of donor gender in FMRP expression was performed using a two-way ANOVA with *post hoc* Sidak’s multiple comparison test.

## RESULTS

**FIGURE 1:**
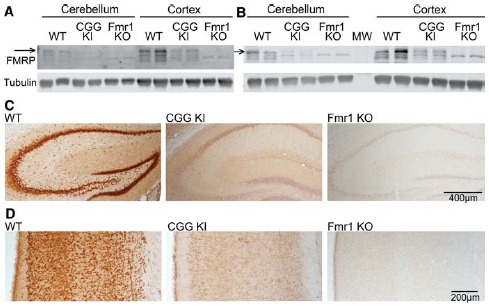
**FMRP antibody specificity. (A)** Mouse α-FMRP 1C3 (Millipore) 1:250 on cerebellar and combined cerebral cortex and subcortical brain lysates of WT (*n* = 2), premutation model CGG KI (*n* = 2), and Fmr1 KO mice (*n* = 2). Arrow indicates the FMRP-specific band which is absent in Fmr1 KO lysates. **(B)** Rabbit α-FMRP 17722 (Abcam) 1:1000 on the same brain lysates as in **(A)**. **(C)** Immunohistochemistry with the 17722 α-FMRP antibody in WT (*n* = 3), CGG KI (*n* = 3) and Fmr1 KO (*n* = 4) hippocampus. **(D)** Cortical FMRP expression from identical animals as in **(C)** stained with the 17722 antibody. Sections were counter-stained with hematoxylin to label nuclei.

To accurately evaluate the expression of FMRP in our experiments, we compared two commonly used antibodies for their specificity by western blot and immunohistochemistry. To assess the sensitivity of each antibody, we compared FMRP levels in WT, littermate CGG KI, and *Fmr1* KO mouse cortical lysates (Figure 1). Both the 1C3 mouse anti-FMRP (Millipore) and the 17722 rabbit anti-FMRP (Abcam) show reactivity in the *Fmr1* KO samples (Figures 1A,B). The largest band at ∼75 kD corresponds to FMRP and is absent in *Fmr1* KO lysates with both antibodies. This band was used for all measurements in subsequent figures. However, a smaller band at ∼71 kD which results at least partially from cross-reactivity with the related protein, FXR1 (Tamanini et al., 1997; Ceman et al., 1999), is still reactive with both antibodies tested. Similarly, we examined specificity of the 17722 anti-FMRP antibody in WT, CGG KI and *Fmr1* KO coronal brain sections by immunohistochemistry (WT *n* = 3, CGG KI *n* = 3, *Fmr1* KO *n* = 4; representative images; Figures 1C,D). Conditions were optimized to minimize DAB reactivity in Fmr1 KO tissue. We reliably observed reduced FMRP levels in CGG KI mice both by western analysis and immunohistochemistry, consistent with previous reports (Tassone et al., 2000a; Kenneson et al., 2001; Entezam et al., 2007; Qin et al., 2011; Iliff et al., 2013; Ludwig et al., 2014; Pretto et al., 2014; Figure 1).

**FIGURE 2:**
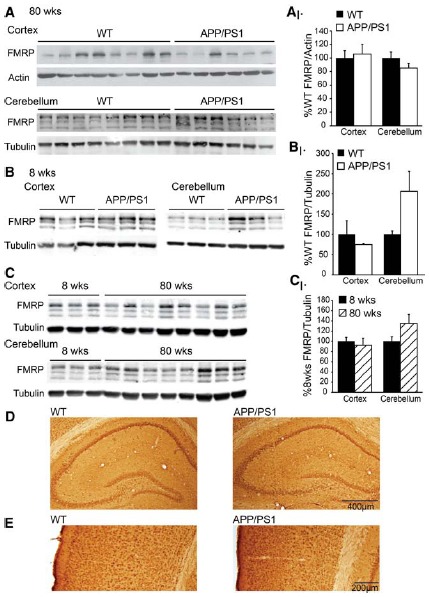
**FMRP expression in AD model APP/PS1 mice. (A)** Cortical and subcortical, and cerebellar lysates from 80-week-old APP/PS1 (*n* = 6) and age-matched controls (*n* = 8) probed with the 17722 α-FMRP antibody and actin or tubulin. **(A1)** FMRP/actin values were calculated and normalized to WT levels, and expressed as %WT in the quantification. Results are the summary of values calculated in technical triplicate. **(B)** Eight-week-old APP/PS1 (*n* = 3) and WT littermate controls (*n* = 3) were compared for FMRP expression in cortical and subcortical and cerebellar lysates. **(B1)** FMRP/tubulin values are expressed as %WT, and data are the summary of experiments performed in technical triplicate. **(C)** Eight-week-old WT (*n* = 3) and 80-week-old WT (*n* = 8) cortical and cerebellar lysates compared for FMRP expression. **(C1)** FMRP/tubulin values are expressed as percentage of mean 8-week-old samples, and data are the summary of experiments performed in duplicate. **(D)** Hippocampal FMRP expression using the 1C3 α-FMRP antibody in WT and APP/PS1 mice. **(E)** Cortical FMRP in the same mice as in **(D).**

In an effort to probe a potential role of altered FMRP expression in AD pathogenesis, we evaluated FMRP in a double transgenic model of AD which contains an additional copy of the human *APP* gene carrying the familial Swedish (K670N/M671L) missense mutation (Haass et al., 1995), and a deletion of exon 9 in the presenilin1 gene (Prihar et al., 1999). These double transgenic mice (APP/PS1; *n* = 6: 6 females) and age-matched control littermates (*n* = 8: 4 males, 4 females) were compared at 80–90 weeks of age for combined cortical and subcortical FMRP levels. We performed western blot analysis in triplicate, averaging the percent control FMRP for each animal across blots to minimize error between experiments. We find no difference in FMRP levels in APP/PS1 mice compared to controls (*t* = 0.358, df = 12, NS; Figure 2A). Cerebellar samples from the same animals demonstrated no significant difference in FMRP expression (*t* = 1.300, df = 12, NS; Figure 2A). Any contribution of sex was evaluated in the control animals (as the APP/PS1 mice were all female), and FMRP expression was not significantly different between male and female control cortical values (male *n* = 4, female *n* = 4, *t* = 0.717, df = 6, NS; data not shown). As several groups have found FMRP levels change with age (Singh et al., 2007; Iliff et al., 2013; Gaur and Prasad, 2014; Ludwig et al., 2014), we explored expression in young (8-week-old) APP/PS1 mice (WT *n* = 3: 3 females; APP/PS1 *n* = 3: 3 females). We find no difference in cortical FMRP expression at this age (*t* = 0.919, df = 4, NS). Cerebellar FMRP expression demonstrated a non-significant increase in AD model mice (*t* = 2.627, df = 4, NS; Figure 2B). We also compared age-dependent FMRP expression of the 8-week-old and the 80-week-old animals, and did not find a significant difference in the cortex or the cerebellum (cortex: 8 weeks, *n* = 3 females; 80 weeks *n* = 8, 4 males, 4 females; *t* = 0.342, df = 9, NS; cerebellum: 8 weeks *n* = 3 females; 80 weeks *n* = 8, 4 males, 4 females; *t* = 1.188, df = 9, NS; Figure 2C). There was no significant difference between same sex 8- and 80-week-old female FMRP expression (cortex: 8 weeks female *n* = 3, 80 weeks female *n* = 4, *t* = 1.034, df = 5, NS; cerebellum: 8 weeks female *n* = 3, 80 weeks female *n* = 4, *t* = 1.136, df = 5, NS; data not shown). We went on to probe FMRP levels by immunohistochemistry in 80-week-old control and APP/PS1 mice. Comparing cortex, hippocampus, and subcortical regions using the 1C3 α-FMRP antibody, there were no differences detected (WT *n* = 2, APP/PS1 *n* = 3; Figures 2D,E).

**FIGURE 3:**
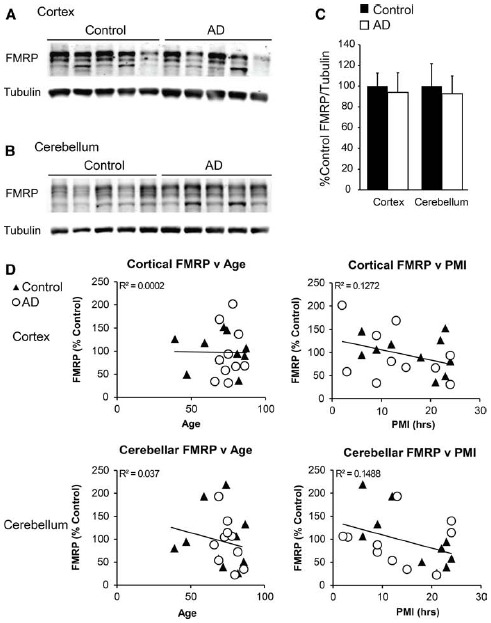
**FMRP expression in Alzheimer’s disease cortex and cerebellum. (A)** Frontal cortex lysates from control (*n* = 10) and autopsy-confirmed AD patients (*n* = 10) probed with α-FMRP 17722. **(B)** Cerebellar lysates from the same patients as in **(A)** were evaluated concurrently. **(C)** Quantification of cortical and cerebellar FMRP/tubulin values performed in technical triplicate. **(D)** Normalized FMRP values were compared to individual patient ages and post-mortem indices. Diagnosis-independent best fit curves display overall trends in FMRP expression.

As the mouse model we used was genetically modified to mimic some AD phenotypes, it may not recapitulate proximal pathogenic events that contribute to spontaneous cases of AD. In an attempt to better answer the question of a possible role for FMRP in AD development, we obtained frontal cortex and cerebellar autopsy samples from control and AD patients (details included in Table 1). As with our murine samples, we performed western blot analysis in technical triplicate to minimize variability in our measurements. Using this technique, we found no difference in FMRP expression level in the frontal cortex (*t* = 0.2836, df = 18, NS; control *n* = 10, AD *n* = 10; Figures 3A,C). As two of the control individuals were younger than the majority of the other donors (39 and 47 years old), we performed analysis excluding those values, and found no change in the result (*t* = 0.3915, df = 16, NS). Similarly there was no difference in FMRP expression found in the cerebellar samples analyzed (*t* = 0.2837, df = 18, NS; control *n* = 10, AD *n* = 10; Figures 3B,C). Again, excluding the youngest individuals did not impact the finding (*t* = 0.3618, df = 16; NS). As there was a wide range in PMI and age of the samples tested, we compared normalized values against these two variables, in addition to tissue pH (where available), and found a significant impact on FMRP levels with longer PMI (age: *r* = –0.111, df = 38, NS; PMI: *r* = –0.371, df = 38, *P* < 0.05; pH: *r* = 0.277, df = 22, NS; Figure 3D, data not shown). We also evaluated the impact of gender in this experiment by comparing the data with a two-way ANOVA, and found no significant difference of FMRP expression in any group in the cortex [gender: *F*_(1,16)_ = 0.3187, NS; diagnosis: *F*_(1,16)_ = 0.05139, NS; interaction: *F*_(1,16)_ = 0.02455, NS; data not shown]. Cerebellar samples similarly showed no significant impact of gender [gender: *F*_(1,16)_ = 0.1176, NS; diagnosis: *F*_(1,16)_ = 0.09488, NS; interaction: *F*_(1,16)_ = 0.006407, NS; data not shown]. While these analyses only showed significant contributions of PMI on FMRP levels, the negative trends of decreasing FMRP with age and lower pH suggest that these variables should also be controlled for in future studies.

**FIGURE 4:**
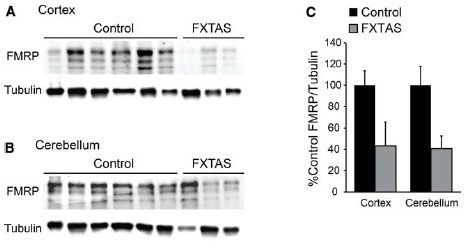
**Cortical and cerebellar FMRP expression in FXTAS patients. (A)** Frontal cortex lysates from FXTAS patients (*n* = 3) and the same control tissues (*n* = 8) probed with α-FMRP 17722. **(B)** Cerebellar lysates from the same individuals as in **(A)**. **(C)** Normalized FMRP expression as a percent of controls, performed in technical triplicate.

We obtained samples from FXTAS patient brains, and compared FMRP levels to the controls, finding a decrement in FMRP immunoreactivity in two of the three samples tested, though the number of samples evaluated was not sufficient to discern a significant difference when the two youngest control samples were excluded (cortex: *t* = 2.161, df = 9, NS; cerebellum: *t* = 1.793, df = 9, NS; control *n* = 8, FXTAS *n* = 3; Figures 4A–C). Similarly, we compared the age and PMI as a factor which may alter FMRP levels, and found no significant difference of either variable (age: *r* = –0.180, df = 24, NS; PMI: *r* = 0.007, df = 24, NS; data not shown).

**FIGURE 5:**
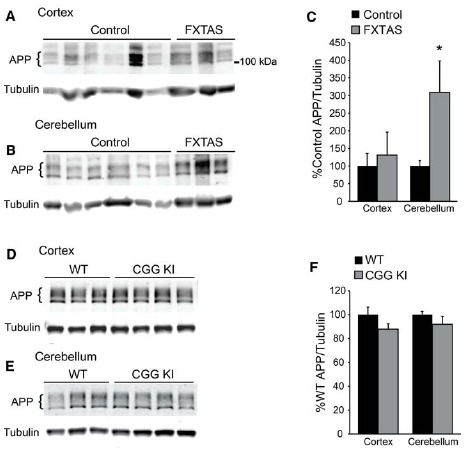
**Amyloid precursor protein expression in FXTAS patients and mouse models. (A)** Frontal cortex lysates from control (*n* = 10) and FXTAS patients (*n* = 3) probed with a C-terminal αAPP antibody which detects the three primary isoforms of full-length APP (100–125 kDa). **(B)** Cerebellar lysates from the same individuals as in **(A)**. **(C)** Normalized APP expression relative to tubulin expressed as a percent of controls, performed in technical triplicate. **(D)** Twelve-month-old CGG KI (*n* = 4) and WT littermate control (*n* = 3) cortex and subcortical lysates probed with αAPP. **(E)** Cerebellar lysates from the same animals as in **(D)**. **(F)** Normalized APP expression in CGG KI mice expressed as a percent of WT controls, performed in technical triplicate. *P < 0.05 Student’s t-test.

To further test the hypothesis that reduced FMRP might enhance APP synthesis, we evaluated total full-length APP expression in the same FXTAS patient samples. Despite the small sample size, we did find a selective increase in cerebellar APP expression in FXTAS samples (*t* = 4.704, df = 11, *P* < 0.05; control *n* = 10, FXTAS *n* = 3; Figures 5A–C). However, APP levels were unchanged in frontal cortex samples compared to controls (*t* = 0.4603, df = 11, NS; control *n* = 10, FXTAS *n* = 3; Figures 5A–C). These results were not affected by the inclusion of the two youngest control samples (cortex: *t* = 0.2636, df = 9, NS; cerebellum: *t* = 4.844, df = 9, *P* < 0.05). This finding was not recapitulated in 12-month-old CGG KI mice, which showed unchanged levels of APP in both the cortex and cerebellum (cortex: *t* = 1.877, df = 5, NS; cerebellum: *t* = 1.131, df = 5, NS; WT *n* = 3, CGG KI *n* = 4; Figures 5D–F). To assess any age-dependent effects of APP expression we compared CGG KI and WT cortical and cerebellar lysates at 2 and 16–18 months of age, and found no significant difference between genotypes (2 months cortex: WT = 100 ± 11.66, CGG KI = 99.86 ± 21.63; *t* = 0.006, df = 8, NS; WT *n* = 5, CGG KI *n* = 5; 16–18 months cortex: WT = 100 ± 15.31, CGG KI = 93.09 ± 6.1; *t* = 0.367, df = 5, NS; WT *n* = 4, CGG KI *n* = 3; 16–18 months cerebellum: WT = 100 ± 10.84, CGG KI = 75.75 ± 7.70; *t* = 1.690, df = 5, NS; WT *n* = 3, CGG KI *n* = 3; data not shown). The C-terminal APP antibody used cannot detect Aβ fragments, so while total APP levels are unchanged, the relative processing or cleavage events may be altered.

## DISCUSSION

FMRP is a synaptic mRNA binding protein implicated in fragile X spectrum disorders. FMRP normally acts to inhibit translation of target transcripts until synaptic transmission and mGluR activation cause its dissociation, allowing for a burst of rapid local translation. One FMRP target is APP, the precursor to the amyloidogenic Aβ peptide (Westmark and Malter, 2007). APP expression is enhanced in mouse models that lack FMRP (Westmark and Malter, 2007). FMRP expression is reported to decline with age in mouse models and might follow a similar trajectory in humans (Singh et al., 2007; Gaur and Prasad, 2014; Ludwig et al., 2014). We hypothesized that a natural age-related decline in FMRP might trigger an increase in basal APP synthesis in neurons, leading to enhanced Aβ-42 production and possibly serving as a proximal trigger of AD pathogenesis. Moreover, as AD pathogenesis progresses, we hypothesized that neuronal dysfunction and toxicity might further impair FMRP expression and hence enhance APP translation, creating a feed-forward loop that could drive AD pathogenesis and lower FMRP expression in AD models and patient tissues. To test this hypothesis, we examined FMRP expression in a double transgenic mouse model of AD (APP/PS1), and found no significant difference between AD model and control animals. Furthermore, we tested control and AD patient samples, and found no difference in cortical or cerebellar FMRP expression. In contrast, we observed a decrease in FMRP levels in the two of the three FXTAS patients tested, consistent with published results (Ludwig et al., 2014; Pretto et al., 2014), and found increased cerebellar levels of APP in these same FXTAS patients, suggesting an impact of lower FMRP on APP expression in humans. Taken together, these data indicate that decreased FMRP expression is not a common finding in AD and suggests that a primary deficiency in FMRP expression is unlikely to play a proximal role in most cases of AD. However, manipulation of FMRP expression and activity retains the potential to influence APP expression and aspects of AD pathogenesis.

FMRP’s regulatory function is dependent on phosphorylation by S6K, and dephosphorylation by PP2A (Narayanan et al., 2007, 2008). Recent work has described a function for FMRP degradation upon mGluR activation, and dephosphorylation by PP2A (Nalavadi et al., 2012). While the goal of this study was to examine total FMRP expression levels, alterations in FMRP phosphorylation would impact its regulatory function, and therefore the control of target protein translation. While we did not observe large alterations in FMRP levels, it is possible that relative phosphorylation state could play a role in AD pathogenesis. Further studies comparing phospho-FMRP would be required to examine this possibility.

Our group and others have observed decreases in FMRP expression with age (Singh et al., 2007; Iliff et al., 2013; Gaur and Prasad, 2014; Ludwig et al., 2014). However, we observed no significant difference in FMRP levels between 8- and 80-week-old WT mice evaluated in this study (Figure 2C). The reason for this difference is not immediately clear, although the smaller number of young animals evaluated and the significant variance observed in older animals may explain the discrepancy. Other groups have evaluated various time points ranging from neonate to adult (20 weeks of age; Singh et al., 2007; Gaur and Prasad, 2014; Ludwig et al., 2014), and as old as 60–70 weeks of age (Singh et al., 2007; Gaur and Prasad, 2014). Both groups have observed cerebellar expression to steadily decrease in older animals (Gaur and Prasad, 2014; Ludwig et al., 2014), though cortical FMRP expression may be more complex during postnatal development into adulthood (Ludwig et al., 2014). While we and others have studied animals near 4–6 weeks of age (Singh et al., 2007; Iliff et al., 2013; Gaur and Prasad, 2014; Ludwig et al., 2014), the specific 8-week time point utilized here has not been compared directly to old animals previously.

Our study is likely underpowered to detect subtle changes in human FMRP expression. We observed a wide range of FMRP expression levels in both the control and patient samples, consistent with published results (Ludwig et al., 2014; Pretto et al., 2014). This variance was not completely explained by patient age, tissue pH, or PMI (Figure 3), although all of these variables demonstrated a trend toward lower FMRP. This leaves open the possibility that lower basal or activity dependent FMRP expression could still contribute to altered APP expression in a subset of patients or in certain brain regions.

Impaired FMRP expression in FXTAS model mice and patients have been described previously (Tassone et al., 2000a; Kenneson et al., 2001; Entezam et al., 2007; Qin et al., 2011; Iliff et al., 2013; Ludwig et al., 2014; Pretto et al., 2014). Similarly, we find reduced FMRP levels in two of the three FXTAS patient samples tested (Figure 4). Symptomatic FXTAS patients can develop cognitive impairment in addition to motor behavior symptoms (Berry-Kravis et al., 2007; Leehey, 2009). While early studies suggested that pathologic hallmarks of AD are rare in FXTAS (Greco et al., 2002), recent studies in female premutation carriers with dementia demonstrated plaque and neurofibrillary tangle development consistent with AD pathology (Tassone et al., 2012). However, a recent study examining AD patient populations for *FMR1* CGG expansions did not find a significant association (Hall et al., 2014).

To test whether FMRP insufficiency might lead to increased APP synthesis in FXTAS, we evaluated APP levels in our patient samples (Figure 5). We detected an increase in cerebellar, but not cortical APP expression in FXTAS patients and no differences in a mouse model of FXTAS (Figure 5). The C-terminal antibody used for this study does not detect Aβ fragments, so it remains possible that APP processing is altered in FXTAS or CGG premutation models. However, in the context of the published studies noted above, a strong direct relationship between CGG repeats and induction of amyloid pathology appears unlikely.

The group 1 mGluR, mGluR5, has been identified as a receptor of the APP peptide Aβ42 (Renner et al., 2010; Sokol et al., 2011; Um et al., 2013; Hamilton et al., 2014). A recent report explored the role of increased mGluR5 signaling in the double transgenic APP/PS1 model of AD (Hamilton et al., 2014). Genetic modulation of mGluR5 improved behavioral learning performance, and decreased amyloid plaques found in the APP/PS1 mice (Hamilton et al., 2014). Furthermore, cortical FMRP expression was increased in 12-month-old APP/PS1 mice compared to WT controls, presumably due to increased mGluR5 signaling, which generates a positive feedback loop leading to increased APP production and cleavage (Hamilton et al., 2014). The reason for differing results in our study is not entirely clear, however, the animals examined in this report were significantly older (18 months old compared to 12 months old), and the procedures for tissue isolation and lysate generation were different (Hamilton et al., 2014). Of note, the increase observed in Hamilton et al. (2014) is consistent with our observations of human AD cerebellar samples which showed increased FMRP expression compared to controls (Figure 3).

While reduced FMRP might contribute to neuronal dysfunction in FXTAS patients, it is unlikely to be a primary cause of neurodegeneration in FXTAS. Expression of CGG repeats as RNA in heterologous contexts is sufficient to elicit toxicity in *Drosophila*, cells, and mice (Jin et al., 2003, 2007; Hagerman and Hagerman, 2004; Iwahashi et al., 2006; Sofola et al., 2007; Hashem et al., 2009; Sellier et al., 2010, 2013; Todd and Paulson, 2010; Renoux and Todd, 2012; Hagerman, 2013). However, FMRP insufficiency may nonetheless contribute to the cognitive decline observed in mouse models and patients with FXTAS. In addition to the potential differential amyloidogenic burden, reduced FMRP likely impacts synaptic function in premutation mouse models (Hunsaker et al., 2012; Iliff et al., 2013; von Leden et al., 2014). Synaptic dysregulation is thought to precede neurodegeneration, and contribute to the onset of symptoms prior to gross neuronal loss (Dong et al., 2009; Milnerwood and Raymond, 2010). Delineating the role of FMRP insufficiency in FXTAS thus remains an important objective going forward given the implications for therapeutic development in patients.

In summary, we find no evidence to support a direct link between lower basal FMRP expression and AD pathogenesis. Future work will be needed to define whether changes in FMRP activity influence AD development, given the known roles of FMRP in APP processing and neuronal function.

### Conflict of Interest Statement

The authors declare that the research was conducted in the absence of any commercial or financial relationships that could be construed as a potential conflict of interest.
